# The impact of ambient temperature on frailty progression in older adults: Evidence from a longitudinal study in China

**DOI:** 10.3389/fpubh.2025.1507400

**Published:** 2025-06-03

**Authors:** Xin He, Zhangbo Cheng, Hua Cao

**Affiliations:** Shengli Clinical Medical College of Fujian Medical University, Fuzhou, Fujian, China

**Keywords:** frailty progression, ambient temperature exposure, older adults, longitudinal cohort study, temperature thresholds

## Abstract

**Background:**

The aging population and frailty-related diseases pose significant public health challenges. This study examined the relationship between ambient temperature and frailty progression in older adults using data from the China Health and Retirement Longitudinal Study (CHARLS).

**Materials and methods:**

Data from 6,187 participants (2015–2018) were analyzed using a standardized Frailty Index (FI). Participants were categorized into the Frailty Progress Rapid Group (FPRG) and Non-Frailty Rapid Progression Group (NFPRG) based on FI changes. Temperature data from 121 Chinese cities were analyzed using logistic regression and subgroup analyses to explore potential modifiers.

**Results:**

The Lowest Daily Average Temperature (TLDAT) and Average Annual Temperature (AAT) showed a negative association with frailty progression. The relationship between The Highest Daily Average Temperature (THDAT) and frailty progression was non-linear, with a turning point at 31.8°C. Subgroup analyses revealed that higher THDAT had a stronger impact on frailty progression in individuals with lower education and those living in rural areas.

**Conclusion:**

Older adults benefit from environments with a TLDAT above −9°C, a THDAT below 31.8°C, and an AAT above 17°C. Public health strategies should consider temperature thresholds alongside sociodemographic factors like education and residence, which influence frailty progression.

## 1 Introduction

The global acceleration of population aging has introduced significant socioeconomic challenges, with age-related health conditions placing an especially heavy burden on healthcare systems (1, 2). Among various geriatric syndromes, frailty—defined as a state of diminished physiological reserve and increased vulnerability to external stressors—has emerged as a critical predictor of adverse health outcomes (3). This multidimensional construct encompasses limitations in activities of daily living, physical functioning, chronic disease burden, and mental health status (4). The Frailty Index (FI), originally conceptualized by Rockwood and Mitnitski (5) through the deficit accumulation model, is now widely recognized as the gold standard for assessing frailty severity. By systematically aggregating health deficits across diverse domains, the FI facilitates precise risk stratification and enables longitudinal monitoring of functional decline in older adults (6, 7).

While genetic predisposition and clinical comorbidities undoubtedly contribute to frailty development, accumulating evidence highlights environmental factors as modifiable determinants (8–10). Recent epidemiological studies have demonstrated associations between air pollutants—including PM2.5, nitrogen dioxide (NO_2_), and ozone—and accelerated declines in physical function (11, 12). Notably, the Global Burden of Disease Study identified non-optimal ambient temperatures as a major contributor to global mortality, particularly among older populations (13). Although seminal work by Mou et al. (10) revealed links between extreme temperatures and cardiometabolic multimorbidity, important knowledge gaps remain regarding the long-term effects of ambient thermal exposure on frailty progression (10). Existing research has largely concentrated on acute temperature–mortality relationships, leaving the chronic impact of temperature on functional capacity insufficiently explored.

To address this gap, we conducted a nationwide longitudinal study using data from the China Health and Retirement Longitudinal Study (CHARLS) from 2015 to 2018. By integrating high-resolution meteorological data from 121 Chinese cities with detailed health assessments. We aimed to: (1) quantify the association between ambient temperature (including extreme lows, highs, and annual averages) and frailty progression; (2) identify optimal thermal thresholds for promoting healthy aging; and (3) evaluate whether sociodemographic characteristics modify these associations. Our findings provide critical insights for developing climate-responsive public health strategies aimed at preserving functional independence in aging populations.

## 2 Method

### 2.1 Data source and participants

Data used in this study were obtained from the China Health and Retirement Longitudinal Study (CHARLS), a nationally representative longitudinal cohort initiated by Peking University in 2011. The study targets the middle-aged and older adults population in China and employs a multi-stage, stratified cluster sampling strategy. Survey content includes psychological health, chronic disease status, and socioeconomic indicators of participants (14–16). To date, five Waves of data have been collected: 2011 (Wave 1), 2013 (Wave 2), 2015 (Wave 3), 2018 (Wave 4), and 2020 (Wave 5), with response rates consistently exceeding 80%. Ethical approval for data collection was granted by the Biomedical Ethics Review Committee of Peking University (IRB00001052-1015), and all participants provided written informed consent. The CHARLS dataset is publicly available at http://charls.pku.edu.cn/en.

**Figure 1:**
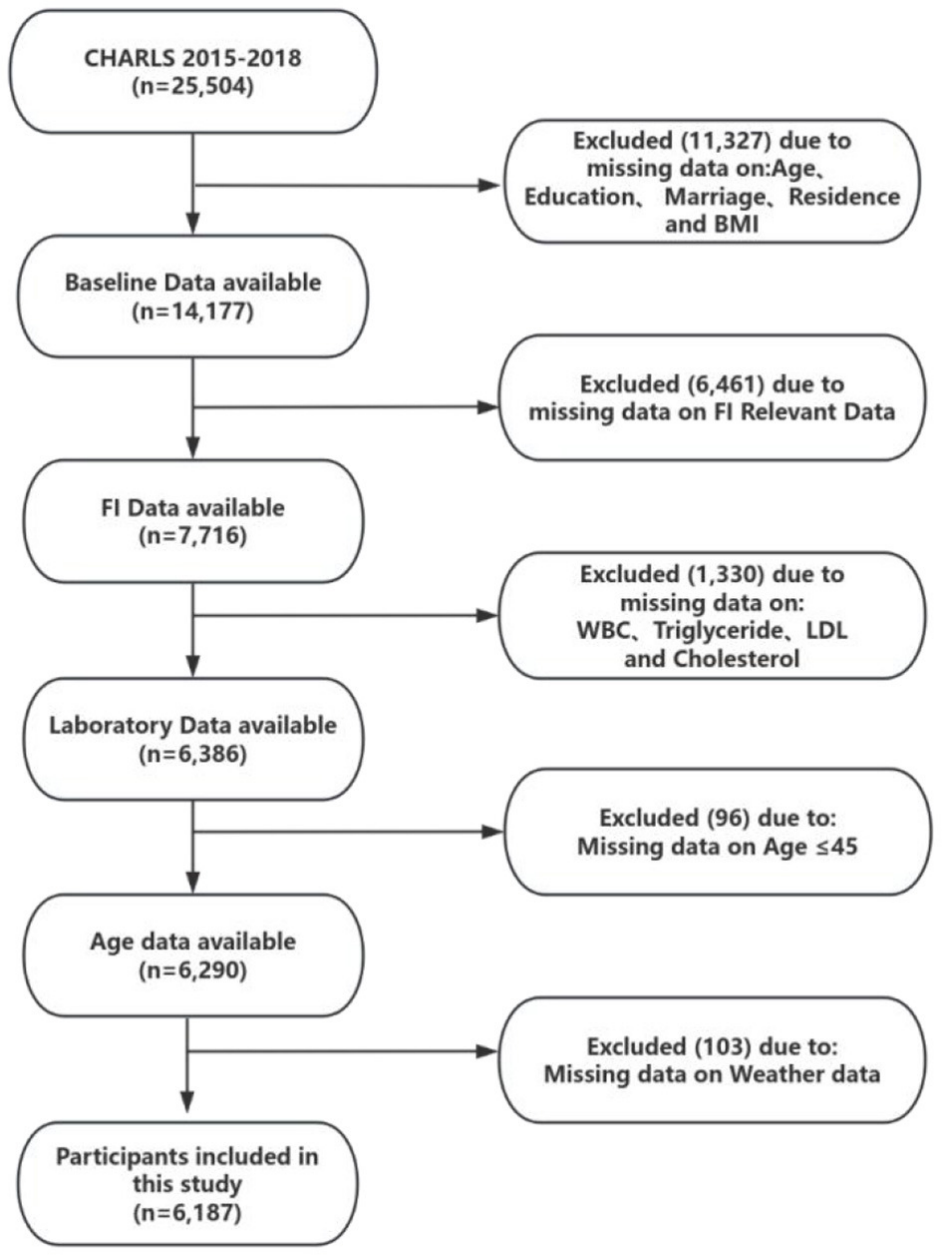
The flow chart of participant selection. CHARLS, China Health and Retirement Longitudinal Study; FI, Frailty Index; WBC, White Blood Cell; LDL, Low Density Lipoprotein.

For this study, we used data from Wave 3 (2015) and Wave 4 (2018). A total of 6,187 participants were included after applying inclusion and exclusion criteria. The detailed screening process is presented in Figure 1. Only participants aged 45 years and above were included, consistent with the CHARLS study design.

### 2.2 Calculation of the FI

The FI used in this study was constructed based on the deficit accumulation model proposed by Rockwood and Mitnitski (5), and further refined using the practical framework introduced by Theou et al. (17). This version of the FI is widely used in geriatric research due to its flexibility, strong predictive validity, and applicability across different datasets and populations. This scale mainly includes 37 variables, including 5 aspects (Activities of daily living Instrumental activities of daily living, Physical functional limitations, Chronic disease, Mental health), These variables can reflect the “deficits” of participants' physical functions from different aspects. According to Theou et al. (17), a frailty index with 30 or more variables across multiple domains ensures robustness and validity. Therefore, 37 variables were chosen based on data availability and domain coverage (17). In addition, the selection of variables was guided by previous FI studies using CHARLS and other large aging cohorts, ensuring comparability and methodological consistency.

We constructed a 37-item Frailty Index (FI) following the deficit accumulation approach, as shown in Supplementary Table S1. Each item was recoded such that “0” indicated no deficit and “1” indicated the presence of a deficit. For variables with ordinal responses (e.g., mental health items), intermediate values of 0.33 and 0.67 were used to reflect partial deficits based on the degree of symptom severity or frequency. Positively framed items such as “feel happy” were reverse-coded to maintain consistency across all items.

The FI score for each participant was calculated as the sum of non-missing item scores divided by the total number of items (*n* = 37), resulting in a continuous variable ranging from 0 to 1. FI values were computed separately for 2015 and 2018. The difference between FI2018 and FI2015 was then used to quantify frailty progression. Participants with an FI increase >0.1 were classified as having rapid frailty progression (Frailty Progress Rapid, FPR), while others were classified as having non-rapid progression (Non-Frailty Rapid Progression, NFRP). These groups are hereafter referred to as the Frailty Progress Rapid Group (FPRG) and the Non-Frailty Rapid Progression Group (NFPRG).

We defined rapid frailty progression (FPR) as an increase in FI >0.1 between 2015 and 2018. This threshold was chosen to reflect clinically meaningful changes in frailty status over a 3-year period. While some prior studies have modeled frailty progression as a continuous outcome [e.g., (18, 19)], or have considered any increase in frailty scale scores as indicative of progression [e.g., (20)]. Our use of a fixed threshold enabled binary classification and clearer group comparisons.

To ensure the robustness of this threshold, sensitivity analyses were conducted using alternative cutoffs (ΔFI > 0.05 and ΔFI > 0.15), and the associations between temperature and frailty progression remained consistent across these definitions, as shown in Supplementary Table S2.

### 2.3 Temperature data

Meteorological data were obtained from the China Meteorological Administration's Land Data Assimilation System (CLDAS v2.0), integrating multi-source observations with high-resolution spatial (0.0625° × 0.0625°) and temporal (hourly) precision. We acquired 4-year daily and annual average temperature records (2015–2018) for participants' residential cities through standardized measurements at municipal meteorological stations. Three temperature metrics were derived: (a) the lowest daily average temperature (TLDAT) across the study period, (b) the highest daily average temperature (THDAT), and (c) the multi-year average annual temperature (AAT). Data completeness exceeded 95%, with limited gaps (<5%) addressed via spatial interpolation from adjacent stations. Geospatial distribution patterns of these thermal metrics across China are illustrated in Supplementary Figures S1–S3, reflecting substantial regional climatic heterogeneity.

### 2.4 Covariates

The covariates included in the regression models were derived from participants' 2015 data and covered three domains: (1) Demographic and socioeconomic factors: age, sex, body mass index (BMI; overweight vs. non-overweight), education level (literacy vs. illiteracy), marital status (married vs. others), place of residence (urban vs. rural), smoking status (yes/no), and alcohol consumption (yes/no); (2) Routine blood parameters: white blood cell count (WBC)and platelet count (PLT); (3) Lipid metabolism indicators: total cholesterol (TC), low-density lipoprotein cholesterol (LDL-C), and triglycerides (TG).

In this study, participants were classified as illiterate or literate depending on whether they had completed formal schooling. Marital status was divided into married and others based on whether the respondent was currently married and cohabiting. Residence was categorized as urban or rural based on the administrative classification recorded in the CHARLS dataset. BMI was grouped into overweight (>24) and non-overweight (≤ 24) categories according to Chinese criteria.

### 2.5 Statistical analysis

Participant characteristics were summarized using appropriate descriptive statistics. Continuous variables were assessed for normal distribution using Shapiro-Wilk tests, with results presented as mean ± standard deviation for parametric data or median (interquartile range) for non-parametric distributions. Categorical variables were reported as frequencies and percentages. Temperature metrics (TLDAT, THDAT, AAT) were initially analyzed as continuous variables, with subsequent quartile categorization (Q1–Q4) applied to variables demonstrating univariate significance (*P* < 0.05). Group comparisons between Frailty Progression Rapid Group (FPRG) and Non-Frailty Rapid Progression Group (NFPRG) employed Student's *t-*tests for normally distributed continuous variables, Wilcoxon rank-sum tests for skewed data, and χ^2^ tests for categorical variables, as detailed in Table 1.

**Table 1:** Baseline characteristics of participants in 2015 (CHARLS).

**Variables**	**Overall (*n* = 6,187)**	**NFPRG(*n* = 5,309)**	**FRPG (*n* = 878)**	***P***	***SMD***
White Blood Cell (× 1000)	5.73 (4.80, 6.90)	5.73 (4.80, 6.90)	5.72 (4.80, 6.97)	0.171	0.083
Platelets (× 10^9^/L)	201 (160, 243)	201 (159, 242)	202 (161, 248)	0.785	0.785
LDL(mg/dL)	101.158 (83.398, 119.884)	101.158 (83.012, 119.691)	102.317 (83.784, 121.911)	0.253	0.041
Triglycerides(mg/L)	116.814 (84.513, 171.681)	115.929 (84.071, 170.796)	123.009 (87.611, 183.186)	0.022	0.083
Total Cholesterol(mg/dL)	181.853 (161.004, 206.178)	181.467 (160.232, 205.792)	185.328 (163.803, 209.653)	0.010	0.094
AAT(°C)	16.903 (13.502, 18.983)	16.903 (13.930, 19.068)	16.356 (12.442, 18.86)	< 0.001	0.139
THDAT (°C)	31.476 (30.760, 32.565)	31.476 (30.760, 32.565)	31.333 (30.730, 32.565)	0.007	0.094
TLDAT (°C)	−7.212 (−15.068,2.454)	−7.212 (−14.540, 2.454)	−7.985 (−15.986,2.454)	< 0.001	0.129
Age(year)	60 (53–66)	59 (52–65)	62 (55–67)	< 0.001	0.254
**Gender**					
Male (%)	3,018 (48.78)	2,661 (50.12)	357 (40.66)	< 0.001	0.191
Female (%)	3,169 (51.22)	2,648 (49.88)	521 (59.34)		
**BMI**					
Non-overweight (%)	3,177 (51.35)	2,731 (51.44)	446 (50.80)	< 0.001	0.019
Overweight (%)	3,010 (48.65)	2,578 (48.56)	432 (49.20)		
**Marriage**					
Others (%)	1,308 (21.14)	1,144 (21.55)	164 (18.68)	0.060	0.072
Married (%)	4,879 (78.86)	4,165 (78.45)	714 (81.32)		
**Residence**					
Urban (%)	5,011 (80.99)	4,285(80.71)	726 (82.69)	0.182	0.051
Rural (%)	1,176 (19.01)	1,024 (19.29)	152 (17.31)		
**Education**					
Illiteracy (%)	3,827 (61.86)	3,200 (60.28)	627 (71.41)	< 0.001	0.236
Literacy (%)	2,360 (38.14)	2,109 (39.72)	251 (28.59)		
**Drink**					
No (%)	3,304(53.40)	2,805 (52.83)	499(56.83)	0.03	0.080
Yes (%)	2,883(46.60)	2,504(47.17)	379 (43.17)		
**Smoke**					
No (%)	3,520(56.89)	2,992 (56.36)	528 (60.14)	0.04	0.077
Yes (%)	2,667 (43.11)	2,317 (43.64)	350 (39.86)		

Baseline characteristics of participants in 2015, stratified by the Frailty Progression Rapid Group (FPRG) and Non-Frailty Progression Rapid Group (NFPRG). Continuous variables are presented as median (interquartile range, IQR), and categorical variables are presented as number (percentage). The statistical significance (P-value) for comparisons between the two groups was calculated using the appropriate tests. Standardized Mean Differences (SMD) are also presented to assess the magnitude of differences between groups. Key variables include white blood cell count (WBC), platelets, low-density lipoprotein (LDL), triglycerides, total cholesterol, and ambient temperature measures (AAT, THDAT, TLDAT). LDL, Low-density lipoprotein; BMI, Body mass index; AAT, Average annual temperature (2015–2018); THDAT, Highest daily average temperature (2015–2018); TLDAT, Lowest daily average temperature (2015–2018).

**Figure 2:**
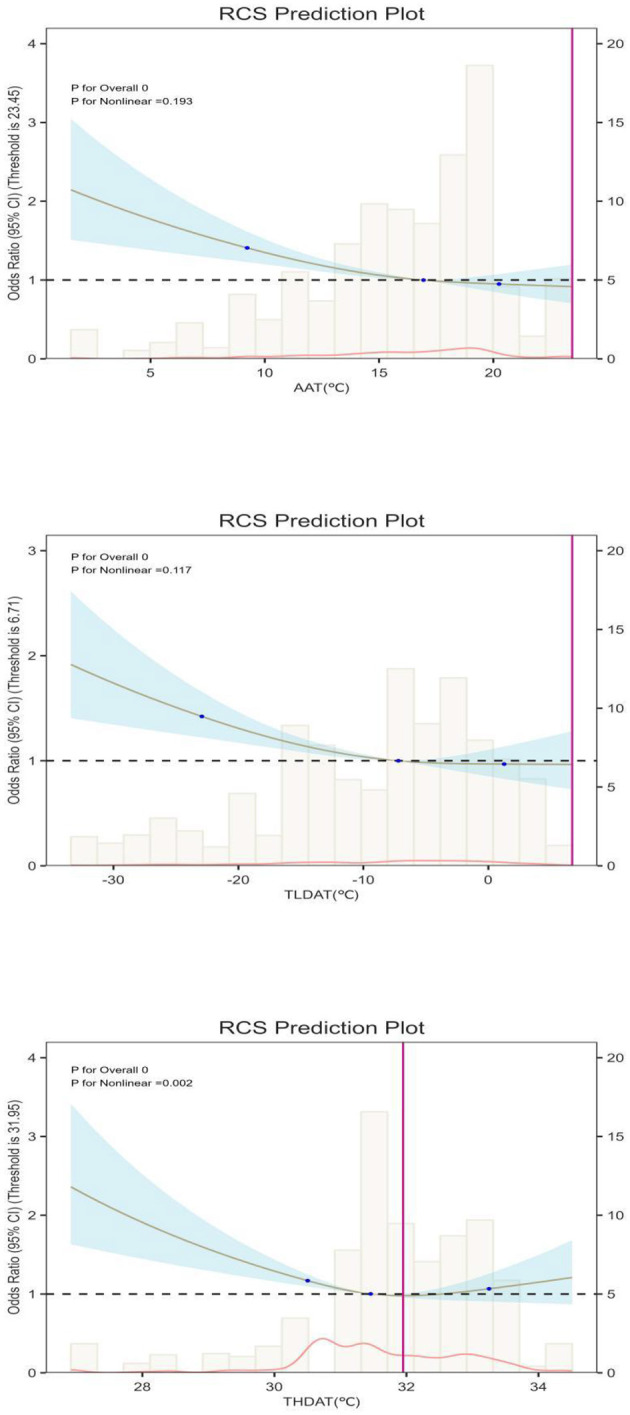
Dose-response association between Weather and FRP. AAT, Average annual temperature (in 4 years); TLDAT, The lowest daily average temperature (in 4 years) THDAT, The highest daily average temperature (in 4 years); X-axis: Ambient temperature (TLM, °C). Left Y-axis: Number of observations (histogram showing temperature distribution). Right Y-axis: Odds ratio (OR) of frailty progression with 95% confidence interval (trend line).

Logistic regression models evaluated associations between temperature metrics and frailty progression risk (as shown in Table 2), calculating adjusted odds ratios (ORs) with 95% confidence intervals (CIs). Three sequential models were developed: (1) unadjusted model; (2) demographic-adjusted model (age, sex, BMI, residence); (3) fully adjusted model incorporating demographic, clinical, and laboratory covariates. Restricted cubic spline regression with three knots (25th, 50th, and 75th percentiles) was employed to explore nonlinear relationships between continuous temperature exposures and outcomes, with the resulting curves illustrated in Figure 2.

**Table 2:** Multivariate logistic regression analyses of Temperature data and FRPG.

**Variable**	**Model 1**	**Model 2**	**Model 3**
	**OR (95%CI)**	**OR (95%CI)**	**OR (95%CI)**
**AAT(**°**C)**			
Continuous (per 1°C)	0.953 (0.934,0.973)	0.955 (0.936,0.975)	0.962 (0.943,0.983)
**Q1 (Reference)**			
Q2	0.717 (0.586,0.876)	0.698 (0.572,0.853)	0.706 (0.579,0.860)
Q3	0.704 (0.575,0.862)	0.709 (0.580,0.866)	0.726 (0.596,0.884)
Q4	0.638 (0.520,0.784)	0.654 (0.534,0.800)	0.706 (0.579,0.860)
*P* for trend	< 0.001	< 0.001	< 0.001
**TLDAT(**°**C)**			
Continuous (per 1°C)	0.983 (0.974, 0.992)	0.984 (0.975, 0.993)	0.987 (0.978, 0.996)
**Q1(Reference)**			
Q2	0.727 (0.594, 0.890)	0.705 (0.577, 0.862)	0.722 (0.592, 0.880)
Q3	0.689 (0.561, 0.847)	0.692 (0.565, 0.848)	0.710 (0.581, 0.866)
Q4	0.704 (0.574, 0.863)	0.720 (0.590, 0.880)	0.776 (0.638, 0.944)
*P* for trend	0.003	0.006	0.005
**THDAT(**°**C)**			
Continuous (per 1°C)	0.936 (0.867, 1.010)	0.942 (0.873, 1.016)	0.953 (0.885,1.027)
**Q1(Reference)**			
Q2	0.978 (0.803, 1.191)	0.966 (0.792, 1.180)	0.994 (0.817, 1.210)
Q3	0.767 (0.622, 0.944)	0.746 (0.606, 0.919)	0.781 (0.636, 0.960)
Q4	0.912 (0.748, 1.112)	0.890 (0.727, 1.090)	0.920 (0.754, 1.123)
*P* for trend	0.088	0.122	0.211

Model 1: unadjusted.

Model 2: adjusted for gender, BMI, education, marriage, residence.

Model 3: adjusted for variables in Model 2 plus age, WBC, Platelets, LDL, Triglycerides, Total Cholesterol, drink and smoke.

AAT, Average Annual Temperature (2015–2018);

TLDAT, Lowest Daily Average Temperature (2015–2018);

THDAT, Highest Daily Average Temperature (2015–2018);

WBC, White Blood Cell; LDL, Low-Density Lipoprotein; Residence: Urban/Rural.

Stratified analyses examined effect modification by sex, BMI status, drink, smoke, education level, marital status, and urban-rural residence. Interaction terms were incorporated into adjusted models, with likelihood ratio tests evaluating subgroup heterogeneity. Sensitivity analyses tested the robustness of the 0.1 FI change threshold using alternative cutoffs (ΔFI >0.05 and >0.15), confirming consistent temperature effects across definitions.

All statistical analyses were performed using R version 4.1.2 (R Core Team, 2021) and SPSS version 26.0(IBM Corp., Armonk, NY, USA). A *P*-value < 0.05 was considered statistically significant, including adjustments for multiple comparisons where applicable (21, 22).

## 3 Results

### 3.1 Participant selection

Our study included 25,504 participants who underwent the baseline (2015) survey. Considering the study objectives, we excluded the following participants: (1) those lacking age information and those under 45 years of age; (2) those lacking information related to FI and other informations at baseline; (3) those who were not followed up;4) those lacking information related to FI and other informations during follow-up surveys. Detailed inclusion and exclusion processes are shown in Figure 1.

To assess potential selection bias, we compared key baseline characteristics between included (*n* = 6,187) and excluded (*n* = 19,317) participants, as shown in Supplementary Table S3. Although some variables showed statistically significant differences (*P* < 0.05)—likely due to the large sample size—their standardized mean differences (SMDs) all remained below 0.1, indicating that the magnitude of these differences was minimal. Consequently, any residual selection bias is expected to be small, and our included sample broadly represents the target population.

### 3.2 Participant characteristics

A comparative analysis of baseline characteristics (Table 1) revealed that participants in the Frailty Progress Rapid Group (FPRG) were generally older (median age 62 vs. 59 years, *P* < 0.001, SMD = 0.254) and had a higher proportion of females (59.34 vs. 49.88%, *P* < 0.001, SMD = 0.191). Although the median BMI was only slightly higher in FPRG (*P* < 0.001, SMD = 0.019), total cholesterol (*P* = 0.010, SMD = 0.094) and triglycerides (*P* = 0.022, SMD = 0.083) were also elevated compared to NFPRG. Additionally, FPRG showed a greater percentage of illiterate individuals (71.41 vs. 60.28%, *P* < 0.001, SMD = 0.236) and had slight but significant differences in drinking and smoking status (*P* = 0.03 and *P* = 0.04, respectively). In contrast, no statistically significant differences were observed for white blood cell count, platelet count, or LDL levels.

From an environmental standpoint, FPRG participants tended to live in regions with significantly lower average annual temperatures (AAT, *P* < 0.001, SMD = 0.139), highest daily average temperatures (THDAT, *P* = 0.007, SMD = 0.094), and lowest daily average temperatures (TLDAT, *P* < 0.001, SMD = 0.129), suggesting that cooler climatic conditions may contribute to accelerated frailty progression.

These findings collectively indicate that older age, female gender, higher lipid profiles, and residence in cooler regions are associated with rapid frailty progression, supporting the notion that both biological and environmental factors can significantly influence frailty trajectories in older adults.

### 3.3 Relationship between temperature data and FPR

Our multivariate analysis revealed significant associations between some thermal exposure metrics and frailty progression (Table 2). After adjusting for all covariates, each 1 °C increase in annual average temperature (AAT) was associated with a 12% reduction in the odds of rapid frailty progression (OR = 0.88, 95%CI: 0.83–0.94), and higher minimum temperatures (TLDAT) likewise conferred a protective effect (OR = 0.91 per 1 °C, 95%CI: 0.86–0.96). Quartile-based analyses for AAT and TLDAT also showed statistically significant, nearly linear trends (*P*-trend < 0.01 for both).

In contrast, for THDAT, the quartile-based trend test was not statistically significant (*P*-trend > 0.05), suggesting no clear linear dose-response across THDAT quartiles. However, restricted cubic spline (RCS) models (Figure 2) revealed a significant non-linear relationship (*P*-nonlinear = 0.008), characterized by a J-shaped curve and a turning point at ~31.8 °C. Below this threshold, each 1 °C increase was associated with a 9% lower risk of frailty progression (OR = 0.91, 95%CI: 0.87–0.96), but above 31.8 °C, the frailty risk rose by 13% per 1°C increase (OR = 1.13, 95%CI: 1.05–1.21). These findings indicate that older adults experience a protective effect of moderate warmth, yet extreme heat may sharply exacerbate frailty progression.

### 3.4 Subgroup analysis

We conducted subgroup analyses to examine whether the association between temperature and frailty progression was consistent across different populations (Supplementary Figures S4–S6). Stratified analyses were performed based on gender, BMI, marital status, residence, drink, smoke, and education level.

The results indicated that the negative associations between AAT and TLDAT and frailty progression remained consistent across most subgroups, with all interaction terms showing *P* > 0.05. This suggests that the effects of these temperature variables on frailty progression were stable across different groups. However, the interaction terms for education level and place of residence with THDAT were significant (*P* < 0.05). Individuals with lower education levels or those living in rural areas were more vulnerable to extreme temperatures, likely due to limited access to climate-responsive resources, while urban populations were less affected.

These findings highlight that the impact of temperature on frailty progression is modulated by factors such as BMI, education level, and residence. Public health interventions should consider these factors to better mitigate the adverse effects of temperature extremes.

## 4 Discussion

As population aging accelerates and climate variability becomes more pronounced, safeguarding the health and functional independence of older adults has become increasingly urgent. Frailty, a multidimensional syndrome characterized by reduced physiological reserve, is particularly sensitive to environmental stressors such as temperature extremes. While numerous studies have linked ambient temperature to mortality and specific chronic conditions in older populations, the long-term relationship between temperature exposure and frailty progression remains insufficiently explored, especially in China. By utilizing nationally representative longitudinal data from the China Health and Retirement Longitudinal Study (CHARLS) and incorporating precise meteorological records, our study offers novel insights into how different dimensions of ambient temperature affect frailty trajectories over time (23).

Temperature data was matched with participant city information in the database. This study primarily extracted baseline data of relevant participants from 2015 and 2018 and quantified the frailty of each participant using the Frailty Index (FI). We quantified the degree of frailty development by calculating the difference in FI between participants in 2018 and 2015. We divided participants into two groups: the Frailty Progress Rapid Group (FPRG) and the Non-Frailty Rapid Progression Group (NFPRG), based on their degree of frailty progression. The differences in relevant indicators between the two groups were then confirmed using statistical methods, which allowed us to explore further the impact of temperature on frailty progression.

We first confirmed through the analysis of baseline data that compared to NFPRG, FPRG participants were older, had higher total cholesterol, higher BMI, and had a higher probability of rapid frailty progression in females and rural populations. This is similar to the results of previous cross-sectional studies, but we have confirmed that these factors are the reasons for the faster progression of the FI index in the middle-aged and older adults (24–26). In terms of temperature data, we obtained the lowest monthly temperature (TLDAT), highest monthly temperature (THDAT), and average annual temperature (AAT) of the participant's city over the past 4 years through investigation. We confirmed through baseline analysis that FPRG had higher TLDAT, THDAT, and AAT.

We transformed the continuous variables THDAT, TLDAT, AAT, and quartiles into level variables, and further confirmed through multiple logistic regression models that these different levels of temperature data have distinct effects on FPR, with statistical significance (*P* < 0.05). Further dose-response analysis indicated a linear relationship (*P* for non-linear > 0.05) between AAT, TLDAT, and FPR. As shown in Figure 2, when AAT exceeds 17°C, excessive AAT has a hazardous effect on FPR in the older adults. Similarly, when TLDAT exceeds −9°C, the protective effect of AAT on the physical “deficiency” of the older adults tends to stabilize. This suggests that the protective effect of temperature on aging bodily functions is effective within a certain range, and when it exceeds this range, the effect diminishes.

In our analysis, THDAT exhibited different patterns compared to AAT and TLDAT. While a quartile-based trend test did not reach statistical significance (*P*-trend > 0.05), the restricted cubic spline (RCS) analysis uncovered a marked non-linear (J-shaped) relationship, with a turning point around 31.8 °C. This discrepancy suggests that simple linear assumptions may fail to capture threshold-like behavior.

Specifically, below 31.8°C, each 1°C increase in THDAT was associated with a 9% reduced risk of rapid frailty progression, indicating that moderate warmth exerts a protective role. However, beyond 31.8°C, the risk rose by 13% per 1°C increment, underscoring the detrimental impact of extreme heat on cardiovascular and cerebrovascular systems in older adults (27, 28).These findings highlight the importance of recognizing non-linear temperature thresholds when developing climate-responsive interventions.

From a public health perspective, our data suggest that maintaining THDAT below 31.8°C, TLDAT above −9°C, and AAT above 17°C may collectively mitigate frailty progression. This aligns with previous evidence linking both cold spells and heat waves to adverse outcomes, further emphasizing the vulnerability of older adults to temperature extremes.

We grouped participants based on gender, BMI, marital status, place of residence, drink, smoke, and education level and confirmed through subgroup analysis that AAT and TLDAT had stable and non-interactive effects on FPR across different groups (*P* for interactions >0.05). Subgroup analysis of THDAT and FPR further suggests that THDAT has a stronger protective effect on participants with normal BMI. For overweight participants, however, THDAT did not significantly affect FPR. This may be because higher fat mass in overweight individuals might help maintain body temperature stability, although the specific mechanism requires further research.

Moreover, the interaction terms for THDAT and education level, as well as place of residence, were statistically significant (*P* < 0.05), suggesting that the protective effects of temperature on frailty progression may differ by these sociodemographic factors. In particular, individuals with lower education levels and those living in rural areas appeared more vulnerable to extreme temperatures. This may be due to limited access to climate-responsive resources and healthcare, further underlining the need for targeted public health interventions for these high-risk groups.

Our research shows that the daily average minimum temperature, daily average maximum temperature, and annual average temperature all influence the progression of frailty in the older adults throughout the year. The impact of cold on the older adult may be due to the fact that middle-aged and older adult people are more sensitive to cold environments. The primary cause of frailty in the older adults is the decline in physical function due to cardiovascular and cerebrovascular diseases. In cold conditions, plasma viscosity increases, and peripheral circulation resistance also rises, which can lead to stroke and ischemic heart disease, further deteriorating physical function (29, 30).

Additionally, studies have shown that the comfortable temperature range for the older adult is higher than that of younger individuals, as their ability to regulate body temperature diminishes, preventing them from maintaining optimal core temperature (31, 32). This change can result in reduced catecholamine secretion, causing mental and psychological dysfunction (33, 34). Our research also highlights that living in rural areas is a disadvantageous factor for frailty development, possibly due to inadequate insulation measures in rural areas. In contrast, urban areas in northern China, with centralized heating systems, provide better protection against the negative impacts of extreme cold weather on the older adult.

The impact of heat waves on the older adults primarily manifests as high temperatures accelerating the evaporation of urban water bodies and increasing urban humidity. These droplets combine with respiratory viruses, accelerating their spread and increasing the incidence of respiratory diseases (35). Furthermore, excessive evaporation of sweat and insufficient hydration in the older adult under extreme high temperatures can lead to strokes (36). High temperatures are also closely linked to cardiovascular diseases, chronic kidney disease, and other conditions (37, 38).

Our research has several advantages. Previous studies have explored the effects of temperature on mortality and cardiovascular and cerebrovascular diseases in the older adults from various perspectives. This study, however, links daily living ability, physical function, mental and psychological disorders, and chronic diseases of the older adults through the Frailty Index (FI). By utilizing the FI, we are able to more comprehensively demonstrate the impact of temperature on the overall physical function of older adults. Furthermore, our longitudinal cohort study allows us to track changes in the FI index over time, providing a better understanding of how temperature affects frailty progression.

## 5 Limitations

This study relied on city-level temperature data rather than individualized measurements, potentially underestimating personal microenvironments—especially for older adults spending variable time indoors. We also did not assess other environmental factors (e.g., humidity, air pollutants), which may interact with temperature and further influence frailty. Although certain baseline characteristics showed *P* < 0.05 when comparing included and excluded participants, standardized mean differences were below 0.1, indicating minimal practical bias. Moreover, frailty is multifactorial; unmeasured lifestyle factors (e.g., diet, physical activity) may have introduced residual confounding. Finally, using a fixed threshold (ΔFI > 0.1) to define rapid frailty progression may overlook more nuanced changes. Future research should employ more granular temperature assessments, account for additional environmental exposures, and consider both continuous and threshold-based definitions of frailty progression.

## 6 Conclusions

This study highlights the significant impact of temperature on frailty progression in older adults. Environments with a TLDAT above −9°C, a THDAT below 31.8°C, and an AAT above 17°C are optimal for delaying frailty progression.

Subgroup analyses show that individuals with lower education and those from rural areas are more vulnerable to extreme heat. These findings emphasize the need for targeted public health interventions that account for both temperature thresholds and sociodemographic factors, to protect older adults from the adverse effects of temperature extremes.

## Data Availability

The original contributions presented in the study are included in the article/Supplementary material, further inquiries can be directed to the corresponding author.
