# Supplementary material for: The impact of ambient temperature on frailty progression in older adults: Evidence from a longitudinal study in China
Source: Front Public Health. 2025 Jun 3;13:1507400. doi: 10.3389/fpubh.2025.1507400 (PMC12170505; doi:10.3389/fpubh.2025.1507400)
Supplement: Supplementary file 1 [file Table_1.docx]

**Table S1. List of health deficits items included in the frailty index**

|  | **Itme** | **Variables** | **Cut-off point** |
| --- | --- | --- | --- |
| Activities of daily living | 1 | Bathing | Difficult=0 Needless help=1 |
|  | 2 | Dressing | Difficult=0 Needless help=1 |
|  | 3 | Use of toilet | Difficult=0 Needless help=1 |
|  | 4 | Continence | Difficult=0 Needless help=1 |
|  | 5 | Eating | Difficult=0 Needless help=1 |
|  | 6 | Geting in/out bed | Difficult=0 Needless help=1 |
|  | 7 | Controling urination and defecation | Difficult=0 Needless help=1 |
| Instrumental  activities of daily living | 8 | Using telephone | Difficult=0 Needless help=1 |
|  | 9 | Managing money | Difficult=0 Needless help=1 |
|  | 10 | Taking medicine | Difficult=0 Needless help=1 |
|  | 11 | Shopping | Difficult=0 Needless help=1 |
|  | 12 | Cooking | Difficult=0 Needless help=1 |
|  | 13 | Doing household | Difficult=0 Needless help=1 |
| Physical functional limitations | 14 | Running or jogging about 1 km | Difficult=0 Needless help=1 |
|  | 15 | Walking 100m | Difficult=0 Needless help=1 |
|  | 16 | Able to stand up from sitting | Difficult=0 Needless help=1 |
|  | 17 | Climbing several flights of stairs without resting | Difficult=0 Needless help=1 |
|  | 18 | Stooping, kneeling, or crouching | Difficult=0 Needless help=1 |
|  | 19 | Lift a weight of 5 kg | Difficult=0 Needless help=1 |
|  | 20 | Able to pick up a coin from a table | Difficult=0 Needless help=1 |
|  | 21 | Reaching or extend arms | Difficult=0 Needless help=1 |
| Chronic disease | 22 | Hypertension | Yes=1; no=0 |
|  | 23 | Diabetes | Yes=1; no=0 |
|  | 24 | Chronic lung diseases (Chronic bronchitis, emphysema) | Yes=1; no=0 |
|  | 25 | CVD | Yes=1; no=0 |
|  | 26 | Stroke | Yes=1; no=0 |
|  | 27 | Emotional, nervous, or psychiatric problems | Yes=1; no=0 |
|  | 28 | Arthritis | Yes=1; no=0 |
|  | 29 | Dyslipidemia | Yes=1; no=0 |
|  | 30 | Liver disease | Yes=1; no=0 |
|  | 31 | Kidney disease | Yes=1; no=0 |
|  | 32 | Gastric or duodenal ulcer | Yes=1; no=0 |
|  | 33 | Asthma | Yes=1; no=0 |
|  | 34 | Memory related disease (Dementia, brain atrophy, and  Parkinson's disease) | Yes=1; no=0 |
| Mental health | 35 | Feel could not get "going" | Yes=1; no=0 |
|  | 36 | Feel happy | Always=0; often=0.33; sometimes=0.67;  seldom or never=1 |
|  | 37 | Feel everything was an effort | Always=1; often=0.67; sometimes=0.33;  seldom or never=0 |
|  | 38 | Feel depressed | Always=1; often=0.67; sometimes=0.33;  seldom or never=0 |
|  | 39 | Feel fearful | Always=1; often=0.67; sometimes=0.33;  seldom or never=0 |

**Table S2 Multivariate Logistic Regression Analysis of Frailty Progression Based on Different Frailty Index (FI) Change Thresholds (0.05, 0.10, 0.15)**

**OR (95% CI, *P*-value)**

| Variable | FI Change Group (0.05) | FI Change Group (0.10) | FI Change Group (0.15) |
| --- | --- | --- | --- |
| AAT(℃) | 0.985(0.973,0.997),  0.016 | 0.962(0.946,0.977),  ＜0.001 | 0.951(0.932,0.971),  ＜0.001 |
| TLDAT(℃) | 0.994(0.988,0.999),  0.032 | 0.982(0.975,0.990),  ＜0.001 | 0.978(0.968,0.988),  ＜0.001 |
| THDAT(℃) | 0.962(0.923,1.002),  0.063 | 0.914(0.866,0.965),  0.001 | 0.891(0.831,0.958),  0.002 |

Multivariate logistic regression analysis evaluating the relationship between ambient temperature and frailty progression, based on Frailty Index (FI) change groups defined by thresholds of 0.05, 0.10, and 0.15. The analysis was adjusted for covariates including age, gender, education level, marital status, platelet count, total cholesterol, triglycerides, white blood cell count (WBC), low-density lipoprotein (LDL), BMI, smoking, and alcohol consumption. Results are presented as Odds Ratios (OR) with 95% Confidence Intervals (CI) and p-values.

**TableS3 Comparative Analysis of Baseline Characteristics Between Included and Excluded Participants**

| **Variable** | **Overall** | **Excluded Group (n=19317)** | **Included Group (n=6,187)** | ***P*** | **SMD** |
| --- | --- | --- | --- | --- | --- |
| n | 25504 | 19317 | 6187 |  |  |
| Age（years） | 58 (50,66) | 57 (48,66) | 60 (53,66) | <0.001 | 0.194 |
| BMI（kg/m^2^） | 23.671 (21.377,26.282) | 23.606 (21.337,26.232) | 23.864 (21.501-,6.459) | 0.071 | 0.018 |
| Gender |  |  |  |  |  |
| Female (%) | 13202 (51.76) | 10033 (51.94) | 3169 (51.22) | 0.332 | 0.014 |
| Male (%) | 12302 (48.24) | 9284 (48.06) | 3018 (48.78) |  |  |
| **Marriage** |  |  |  |  |  |
| Others (%) | 4974 (19.50) | 3666 (18.98) | 1308 (21.14) | <0.001 | 0.054 |
| Married(%) | 20530 (80.50) | 15651 (81.02) | 4879 (78.86) |  |  |
| **Residence** |  |  |  |  |  |
| urban(%) | 20336 (79.74) | 15325 (79.33) | 5011 (80.99) | 0.005 | 0.042 |
| rural(%) | 5168 (20.26) | 3992 (20.67) | 1176 (19.01) |  |  |

Values presented as median (range) for continuous variables and count (%) for categorical variables. *P*-values calculated using Mann-Whitney U test for continuous variables and χ² test for categorical variables. SMD = Standardized Mean Difference


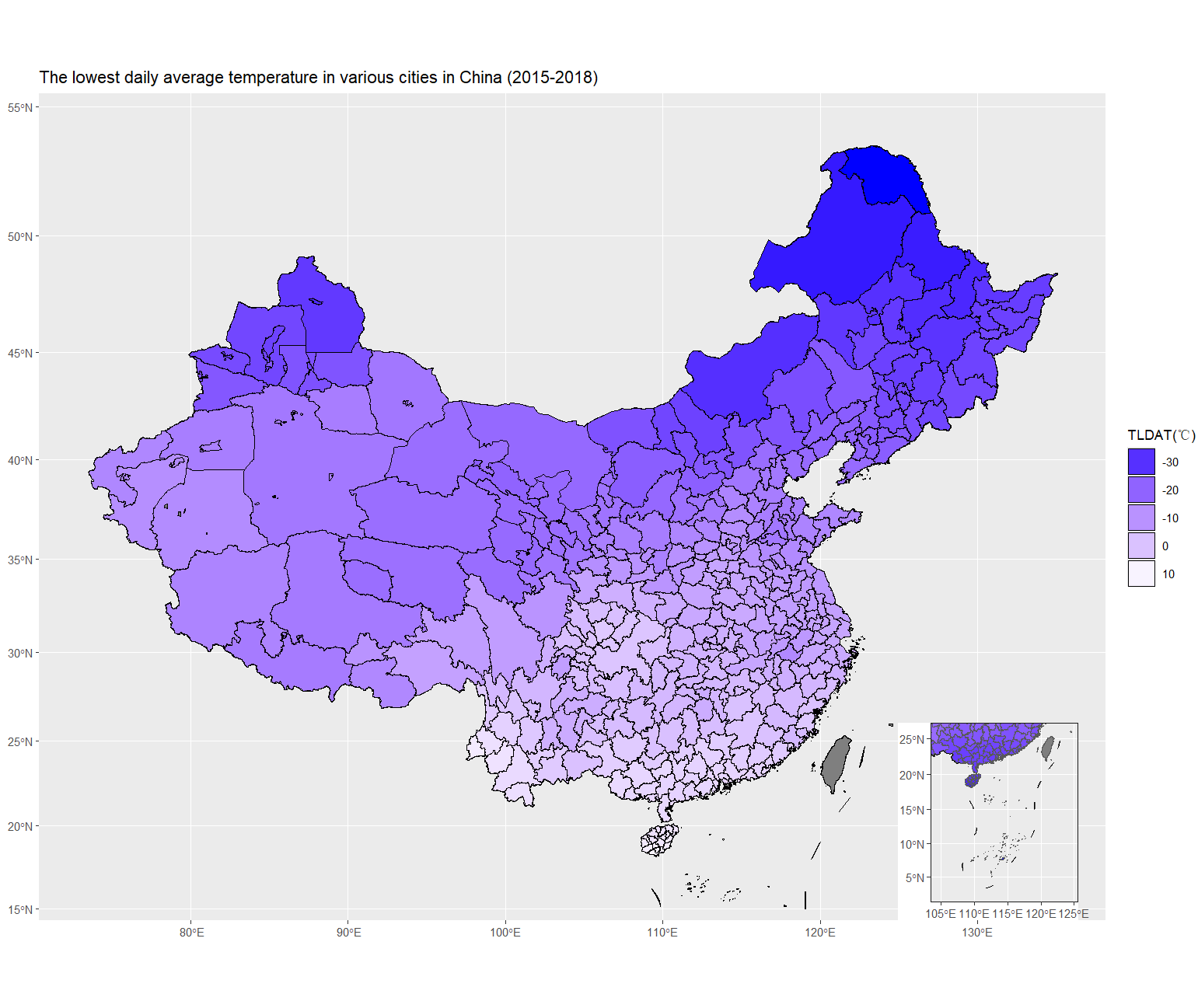


**TableS1 The lowest daily average temperature in various cities in China(2015-2018)(Excluding Taiwan Province)**

**
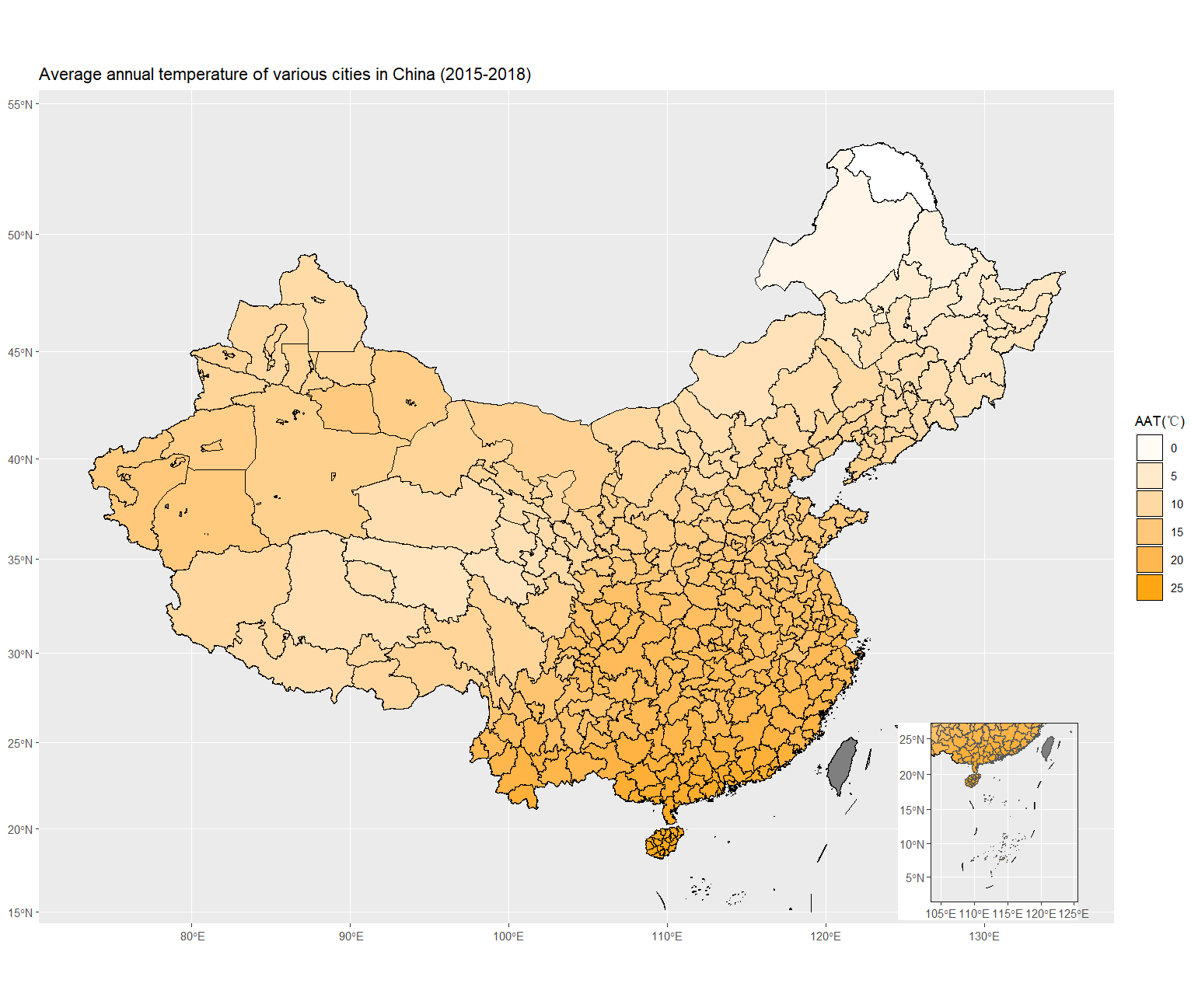
**

**TableS2 Average annual temperature in various cities in China(2015-2018)(Excluding Taiwan Province)**

**
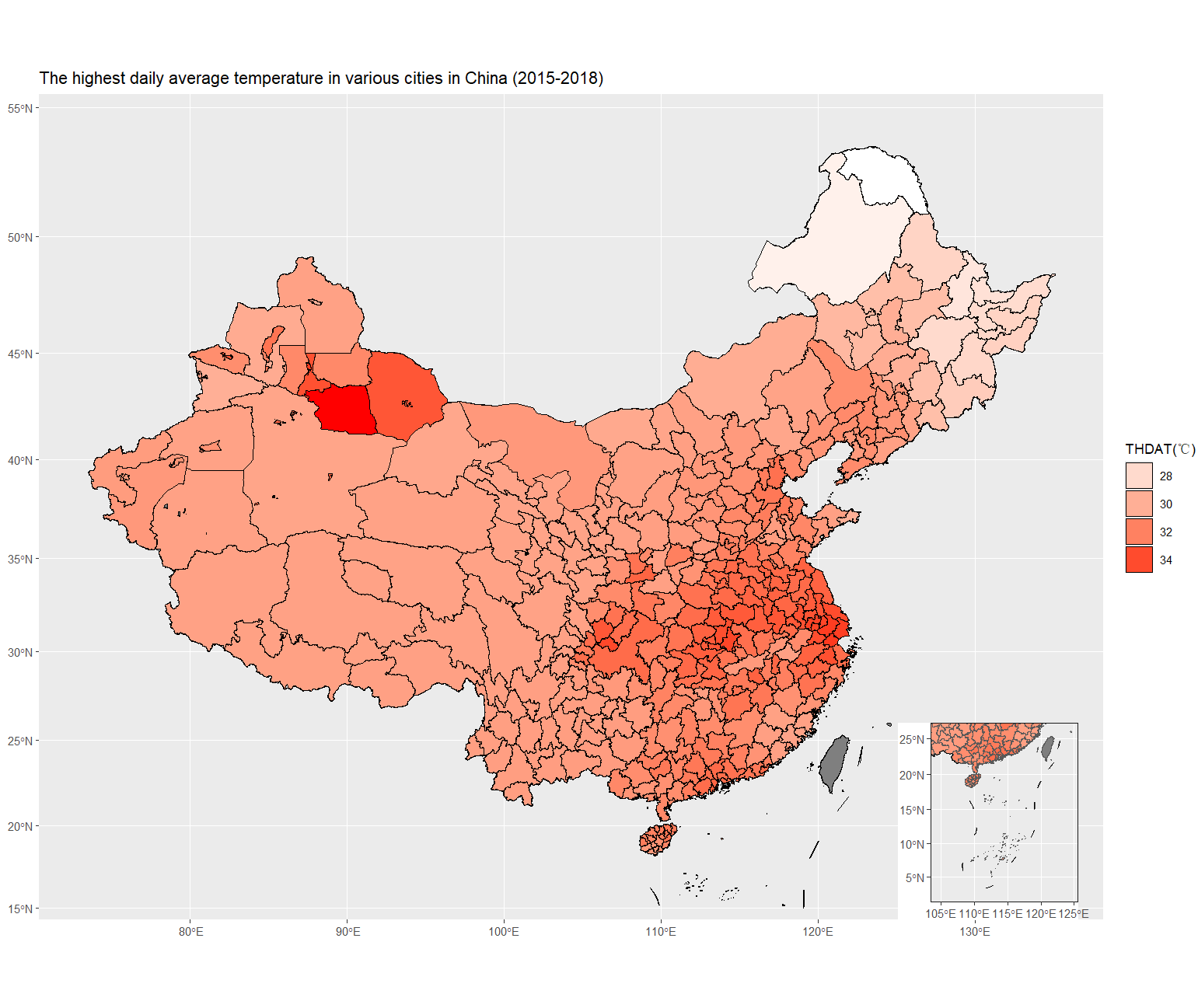
**

**TableS3 The highest daily average temperature in various cities in China(2015-2018)(Excluding Taiwan Province)**


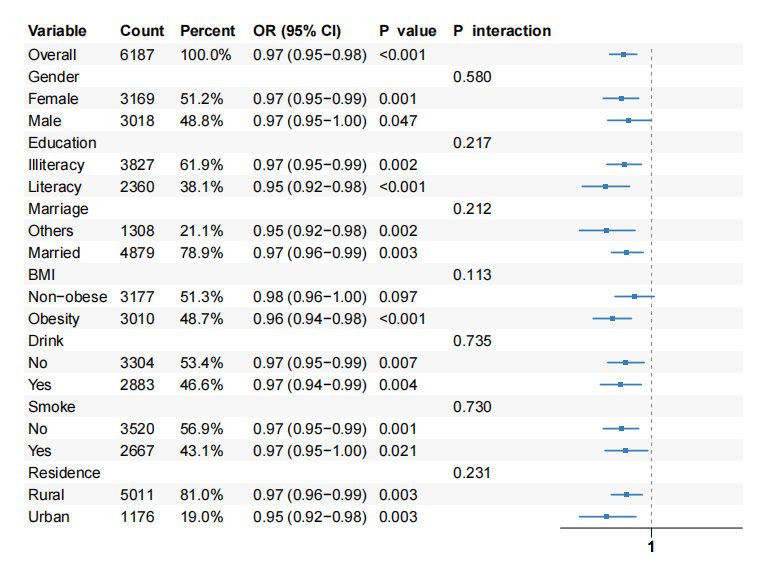


**Figure S4 Subgroup analyses of the association between AAT** **and FRPG**

**AAT: Average Annual Temperature (2015–2018)**


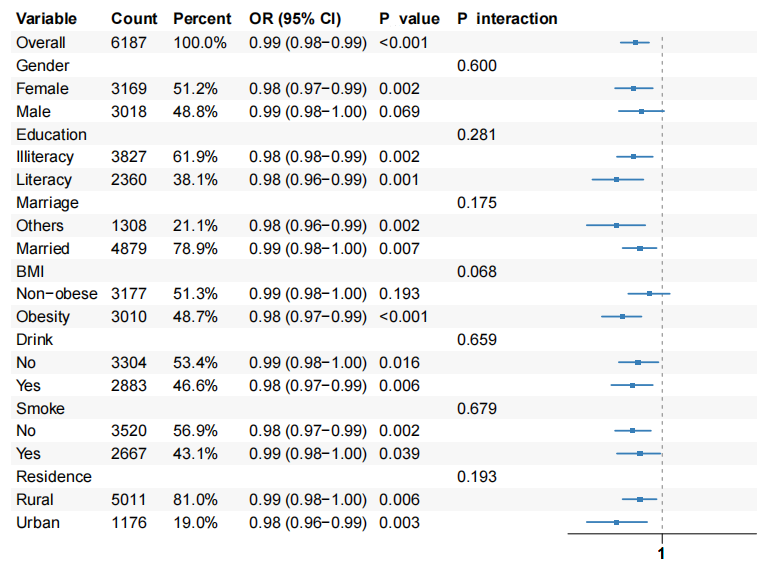


**Figure S5 Subgroup analyses of the association between TLDAT** **and FRPG**

**TLDAT: Lowest Daily Average Temperature (2015–2018)**


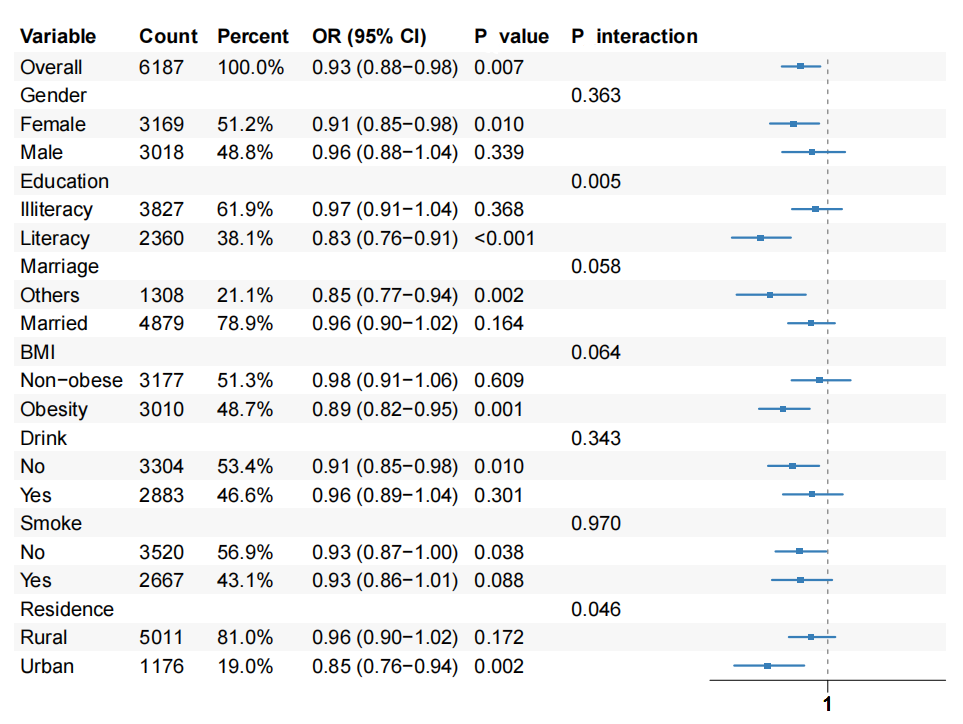


**Figure S6 Subgroup analyses of the association between THDAT and FRPG**

**THDAT: Highest Daily Average Temperature (2015–2018)**
